# Inhibition of miR-222 by Oncolytic Adenovirus-Encoded miRNA Sponges Promotes Viral Oncolysis and Elicits Antitumor Effects in Pancreatic Cancer Models

**DOI:** 10.3390/cancers13133233

**Published:** 2021-06-28

**Authors:** Giulia Raimondi, Sabrina Gea-Sorlí, Marc Otero-Mateo, Cristina Fillat

**Affiliations:** 1 Institut d’Investigacions Biomèdiques August Pi i Sunyer (IDIBAPS), 08036 Barcelona, Spain; g.raimondi90@gmail.com (G.R.); sabrinagea7@hotmail.com (S.G.-S.); motero@clinic.cat (M.O.-M.); 2 Centro de Investigación Biomédica en Red de Enfermedades Raras (CIBERER), 08036 Barcelona, Spain; 3 Facultat de Medicina i Ciències de la Salut, Universitat de Barcelona (UB), 08036 Barcelona, Spain

**Keywords:** miRNAs, oncolytic adenovirus, virotherapy, pancreatic cancer, miRNA-sponges

## Abstract

**Simple Summary:**

Oncolytic adenoviruses are replication-competent viruses engineered for use in cancer treatment. However, the transformation of cancer cells defines a scenario in which the viruses are not adapted to replicate. During tumorigenesis, a large number of miRNAs are deregulated, modulating the expression of genes with essential roles in carcinogenesis and with potential effects on adenoviral propagation. Here we show how OncomiRs can impact adenoviral activity, identifying miR-222 as a limiting factor. Of notice, by genetically engineering a therapeutic adenovirus with miR-222 sponges (AdNuPAR-E-miR222-S), we reduced the latter miRNA content, while enhancing adenoviral fitness, increasing cytotoxicity, and sustaining tumor growth control in xenografts.

**Abstract:**

Oncolytic adenoviruses (OA) are envisioned as a therapeutic option for patients with cancer, designed to preferentially replicate in cancer cells. However, the high number of genetic alterations in tumors can generate a context in which adenoviruses have difficulties replicating. Abnormal miRNAs expression is a trademark of pancreatic cancer, with several oncogenic miRNAs playing essential roles in cancer-associated pathways. The perturbed miRNome induces reprogramming of gene expression in host cells that can impact the complex interplay between cellular processes and viral replication. We have studied the effects of overexpressed miRNAs on oncolytic adenoviral activity and identified miRNAs modulators of adenoviral oncolysis in pancreatic cancer cells. Inhibition of the highly upregulated miR-222 sensitized cancer cells to oncolysis. To provide a therapeutic application to this insight, we engineered the oncolytic adenovirus AdNuPARmE1A with miR-222 binding sites, working as sponges to withdraw the miRNA from the cellular environment. AdNuPAR-E-miR222-S mediated-decrease of miR-222 expression in pancreatic cancer cells strongly improved the viral yield and enhanced the adenoviral cytotoxic effects. Antitumoral studies confirmed a high activity for AdNuPARmE1A-miR222-S in vivo, controlling tumor progression more effectively than the scrambled control virus in xenografts. We demonstrated that the increased antitumor potency of the novel oncolytic virus resulted from the combinatory effects of miR-222 oncomiR inhibition and the restoration of miR-222 target genes activity enhancing viral fitness.

## 1. Introduction

Oncolytic adenoviruses are a promising class of anticancer agents, designed to selectively target and replicate within malignant cells, inducing their lysis and evoking a consistent antitumor immune response [1]. Following T-VEC approval for the treatment of Melanoma, every year more oncolytic products are undergoing clinical development, highlighting their therapeutic potential. Recently, a Phase II study in patients with recurrent high-grade gliomas showed how the intratumoral administration of DNX-2401 resulted in more than 3 years of progression-free survival. Analysis of surgical specimens from those patients documented viral replication and spread within the tumor mass while eliciting a strong immune response against tumor cells [2]. However, despite the promising results, the clinical efficacy of oncolytic adenoviruses remains low, being the lack of potency a major obstacle. Nowadays, several approaches are being explored to enhance the viral activity against tumors, acting either on cancer cells or on viral components. Recently, some attention has been placed on microRNAs (miRNAs). These molecules are small single-stranded (20–25 nt), non-coding RNA sequences, that regulate gene expression at the post-transcriptional level, either by triggering the degradation of target mRNAs, or inhibiting their translation. They participate in a plethora of processes balancing the correct cellular homeostasis and thus having a key role in carcinogenesis. Deregulation of miRNAs expression is a constant characteristic in cancers, which generally show an upregulation of the oncogenic miRNAs (OncomiRs), and a downregulation of the tumor-suppressive ones, favoring the acquisition of cancer hallmarks [3,4]. 

Besides their role in carcinogenesis, miRNAs are also key players in the host-virus interactions, and their levels suffer dramatic changes in the course of an adenoviral infection [5,6]. The alterations in cellular miRNome come both from the necessity of the cell to build a strong antiviral immune response, and from the virus struggling to complete its life cycle [5,7]. Evidence from different studies demonstrated how miRNAs can regulate viral genes, having either a proviral or antiviral function. For instance, host miR-214 binds to the 3' UTR of E1A inhibiting adenoviral replication [8], whereas miR-122 expression is required to complete the hepatitis C virus (HCV) lifecycle [9]. 

Thus, there is a multilayered and complex miRNA-mediated interplay between adenoviruses and host cells, which requires further understanding while trying to improve oncolysis. When adenoviruses infect cancer cells, they face a miRNome that is already perturbed, and that could act against adenoviral replication. In this scenario, the identification of candidate miRNAs able to stimulate viral propagation is necessary but challenging. Until now, functional genetic screenings have centered in tumor-suppressor miRNAs and identified miR-26b, miR-99b, and miR-485 as sensitizers of adenoviral oncolysis [7,10]. However, up to now, no published studies are focusing on the upregulated tumor-promoting miRNAs and their influence on adenoviral oncolysis.

In this work, we studied the role of pancreatic ductal adenocarcinoma (PDAC) OncomiRs on the oncolytic adenoviral activity, identifying miR-222 as a limiting factor for viral propagation. The introduction of miR-222 sponges in an engineered oncolytic adenovirus (AdNuPAR-E-miR222-S) reduced the target miRNAs content, enhanced viral cytotoxicity, and increased viral yields, leading to improved antitumor efficacy in a preclinical model of PDAC.

## 2. Materials and Methods

### 2.1. Cell Lines

The human pancreatic cancer cell lines, PANC-1 and MIA PaCa-2, the human embryonic kidney cell lines, HEK293 and HEK293T, and the lung carcinoma A549 cells were purchased from the American Type Culture Collection (ATCC; Rockville, MD, USA). The human pancreatic tumor cell line CP15-Luc was obtained as previously described [11]. All the cell lines were cultured in Dulbecco’s Modified Eagle’s Medium (Gibco) supplemented with 10% inactivated Fetal Bovine Serum (iFBS), penicillin (100 U/mL), streptomycin (100 µg/mL), and L-glutamine (2 mM). Cells were maintained in a humidified incubator at 37 °C, with 5% CO_2_, and every two months cells were freshly re-plated from the original batch, but not re-authenticated by the authors. Interspecies contamination was routinely tested by PCR detection.

### 2.2. Single Guide RNA Design

sgRNAs were designed with the help of two different software: Benchling (https://www.benchling.com/, accessed on 1 June 2021) and Breaking Cas (https://bioinfogp.cnb.csic.es/tools/breakingcas/, accessed on 1 June 2021) [12]. The sgRNAs were chosen based on high on-target activity and low off-target scores. The sgRNAs were designed as blunted-end sequences to permit ligation in the BsmBI site of the lenti CRISPR v2 vector (Addgene #52961). The sgRNAs were ordered as ssDNA oligos (IDT) and cloned in the lentiCRISPRv2 vector following the protocol described by Zheng Lab (https://media.addgene.org/data/plasmids/52/52961/52961-attachment_B3xTwla0bkYD.pdf, accessed on 1 June 2021). A complete list of the sgRNAs used in the study is available in Table S1. Plasmid constructions were tested by PCR and positive colonies confirmed by Sanger Sequencing (Genewitz), using the specific primer set (LCRISPR-Val, Table S2). 

### 2.3. Generation of CRISPR KO Cell Lines

miRNA-KO cell lines were generated by lentiviral transduction. Lentiviral particles were obtained by calcium-phosphate DNA precipitation transfection of the lenti CRISPR v2 vector containing the sgRNAs, the packaging pCMVAR 8.91, and the envelope pVSV plasmids into HEK 293T cells. Cell supernatant containing lentiviral particles was collected 72h post-transfection and used to infect PANC-1 or MIA PaCa-2 cells. Forty-eight hours later, transduced cells were selected with puromycin (8 µg/mL for PANC-1 and 4 µg/mL for MIA PaCa-2). For PANC-1 miR-21 and miR-93 KO, two different clones were isolated. Alteration in the genomic sequence was analyzed via Sanger Sequencing (Genewitz) using specific primers for each miRNA DNA region (Table S2).

### 2.4. miRNA Sponge Generation

Sponges were generated with the help of the software miRNA song (https://www2.med.muni.cz/histology/miRNAsong/index.php?q=about, accessed on 1 June 2021 (Barta Peskova)). Four consecutive miR-221/222 binding sites interspaced by a 4 nt spacer were selected, and a bulge between the nucleotides 9 and 12 was introduced to increase sponges’ inhibitory effects [13,14,15]. For the Scramble construct, the miR-222 sponge sequence was shuffled to obtain low detection efficiency for miRNAs. Both miR-222 and Scramble sponge sequences were adapted at their 5′ UTR with two extra stop codons (TAA-TGA) to assure the termination of transcription. Sponges extremities were endowed with nucleotides to restore EcoRI recognition sites following cloning into miRVec vector. Sponges were purchased as ssDNA oligos from IDT, resuspended in duplex buffer (IDT) at a final concentration of 100 μM, and annealed by heating at 94 °C for 4 min, followed by a gradual ramp down of temperature. The oligonucleotide sequences are available in Table S3. Annealed oligos were phosphorylated by T4 PNK (NEB) and inserted in the 3′UTR of the EGFP in the miRVec-EGFP plasmid overnight at 4 °C [16]. Plasmid constructions (miRVec-miR222-S and miRVec-Scramble) were tested by PCR using primer set miRVec_Val (Table S2), and positive colonies confirmed by Sanger Sequencing (Genewitz). 

### 2.5. Adenovirus Generation and Titration

pAdNuPAR-E-miR222-S and pAdNuPAR-E-Scramble were generated by an adapted recombineering protocol, consisting of homologous recombination in SW102 bacteria using a positive-negative selection screening with the RpsLNeo cassette [17,18]. The pAdNuPARmE1A genome [11] was modified to express the RpsLNeo cassette in the region between E4 and R-ITR, as previously described [10], generating the pAdNuPARmE1A-RpsLNeo (E4). To clone the sponges inside the pAdNuPARmE1A-RpsLNeo (E4) backbone, the sequences containing CMV-EGFP-miR222-S or CMV-EGFP-Scramble were amplified from miRVEC-miR222-S and miRVec-Scramble, respectively, with specific primers (miRNA-tails, Table S2). The RpsLNeo cassette in the pAdNuPARmE1A-RpsLNeo (E4) backbone was replaced by the amplicons CMV-EGFP-miR222-S or CMV-EGFP-Scramble by homologous recombination. The newly generated pAdNuPAR-E-miR222-S and pAdNuPAR-E-Scramble backbones were validated by PCR (primer set AdV_Val, Table S2) and enzymatic digestion. Once identified positive colonies, those were finally confirmed by Sanger Sequencing. The new adenoviral genomes pAdNuPAR-E-miR222-S and pAdNuPAR-E-Scramble were transfected in HEK293 cells and the viral particles were propagated in A549 cells. Adenoviruses were purified by cesium chloride gradient centrifugation [19] and titrated based on optical density (vp/mL) or viral infectious units (IFU/mL), as previously described [20]. 

### 2.6. Cytotoxicity Assay

CP15-Luc, MIA PaCa-2, and PANC-1 cells were seeded at a density of 5000 cells/well in triplicate. The day after, cells were infected with serial dilutions of the indicated virus (values of dilutions are specified in each graph). Four hours after infection, the medium containing the virus was replaced with a fresh medium (100 µL/well). Viability was measured 7 days later by an MTT colorimetric assay (Affymetrix, USB® Products). Results are expressed as a percentage of viability relative to uninfected control (100%).

### 2.7. Viral Release Assay

PANC-1 and MIA PaCa-2 were seeded at a density of 50,000 and 100,000 cells/well, respectively, in a 24 MW plate. The day after, cells were treated with the indicated amount of virus. Four hours later, cells were washed three times with PBS, to remove viral particles that did not enter cells, and 500 µL of fresh medium were added. Seventy-two hours following infections, cell supernatants containing released viral particles were collected. Viral DNA was extracted both from cells at 4h post-infection and from the supernatants at 72 h, using the Blood DNA Isolation Mini Kit (Norgen Biotek, Thorold, ON, Canada) following the manufacturer's protocol. To quantify adenoviral entry, the number of adenoviral particles/cell was detected by qRT-PCR on a Viia7 (Applied Biosystems, Waltham, MA, USA) using SYBR Green I Master plus mix (Roche, Basel, Switzerland) with specific primers for adenoviral hexon and cellular albumin (Table S4). Only experiments showing an equal amount of genomes entry for the two studied viruses were considered. The viral particles released in the supernatant are expressed as released viral particles/mL (IFU).

### 2.8. Intracellular Viral DNA Quantification

To monitor the amount of viral replicating DNA, PANC-1 cells were seeded at a density of 50,000 cells/well. The day after, cells were infected with 2.5 IFU cells, washed with PBS, and replaced with fresh medium. Infected cells were collected at 4, 16, 20, 24, and 30 h following infection, and viral and cellular DNA was extracted with the Blood DNA Isolation Mini Kit (Norgen Biotek) following the manufacturer’s protocol. The number of viral genomes/cells was calculated as previously described [20]. 

### 2.9. Gene Expression Analysis

Total RNA was extracted from cells and tumors using the miRNeasy Mini Kit (Qiagen). To remove contaminant genomic DNA, samples were treated with DNA-free™ DNA Removal Kit (Invitrogen, Waltham, MA, USA) and quantified at a Nanodrop 1000 spectrophotometer. Five hundred ng of RNA were retrotranscribed to cDNA using the PrimeScript™ RT Reagent Kit (Takara Bio Europe, Saint-Germain-en-Laye, France). mRNA amount was detected by real-time qPCR with the use of specific primers (Table S4) and LightCycler® 480 SYBR Green I Master (Roche) in a ViiA 7 Real-Time PCR System (Applied Biosystems). Reactions were performed in triplicates. Relative gene expression was quantified by the ΔCt method, normalizing individual gene expression to the HPRT gene and represented as 2ΔCT.

### 2.10. miRNA Expression Analysis

Five ng of total RNA was reverse transcribed using TaqMan MicroRNA Reverse Transcription Kit (Applied Biosystem) paired with specific TaqMan stem-loop primers for the desired miRNA (Applied Biosystem), following manufacturer’s instructions. For the qRT-PCR, 1.5 µL of the reverse transcribed miRNA were amplified with TaqMan™ Fast Universal PCR Master Mix no AmpErase™ UNG (Applied Biosystem) and TaqMan microRNA assays specific for the miRNA of interest, as indicated by manufacturers. miRNA expression data were normalized to small nucleolar U6 RNA (RNU6B) and values represented as 2ΔCT. The stem-loop primers and related TaqMan miRNA assays were purchased from Applied Biosystem: RNU6B (001093), hsa-miR-21 (000397), hsa-miR-93-5p (001090), hsa-miR-221-3p (000524), hsa-miR-222-3p (002276).

### 2.11. eGFP Detection and Quantification

eGFP related fluorescence was visualized with an Olympus IX51 microscope at the wavelength of 480 nm. Cells of interest were harvested by trypsinization, and fluorescence intensity was further quantified with flow cytometry, using an AttuneTM Acoustic Focusing Cytometer (Applied Bioscience). Results are expressed as the Mean Fluorescence intensity (MFI).

### 2.12. In Vivo Antitumor Study

Subcutaneous tumors were generated by injecting in each flank of Foxn1nu/nu mice a suspension of 2 × 10^6^ PANC-1 cells/flank. Briefly, cells were harvested by trypsinization and washed twice with PBS by centrifugation. The required number of cells was resuspended in a final volume of PBS and Matrigel 1:1, and 100 µL of this suspension was injected with a 29G needle to generate tumors. Tumors’ volume was monitored with the formula V = (D·d^2^·3.1416)/6, and when the mean reached 100 mm^3^, animals were randomly allocated to treatment groups and intravenously injected either with a single dose of saline solution or 5 × 10^10^ vp/animal of AdNuPAR-E-miR222-S or AdNuPAR-E-Scramble. Tumor volumes were monitored twice a week, and when their dimension reached ethical concerns, animals were euthanized, and tumors were collected for further analysis. 

### 2.13. In Vivo Toxicity Assay

C57Bl/6 mice were treated i.v. with saline solution or a single dose of AdNuPAR-E-miR222-S 5 × 10^10^ vp/animal and body weight was measured for 14 days.

### 2.14. Identification of Putative Targets of hsa-miR-222-3p

We selected a group of upregulated genes from the list provided by Reyes and collaborators having a minimum 2-fold increase during adenoviral infections [21]. From this list, we analyzed with the miRTarBase (https://mirtarbase.cuhk.edu.cn/~miRTarBase/miRTarBase_2019/php/index.php, accessed on 1 June 2021) those that were recognized targets for hsa-miR-222, according to at least one predicted technique. A list of 9 candidates were selected and based on functional data obtained by literature search we finally end up with a shortlist of 5 candidates: BPTF (MIRT046732), DDX21 (MIRT046685), KPNA2 (MIRT474333), NOLC1 (MIRT046772), and TCOF1 (MIRT046740).

### 2.15. Statistical Analysis

Experimental values are represented as mean ± SEM of at least three independent experiments, and each experiment was performed in triplicate. Data were analyzed with the help of the software GraphPad Prism v 8.0.1, and statistical differences were evaluated by the application of a 2-tailed non-parametric Mann–Whitney test, and *p* < 0.05 was taken as the level of significance.

For the in vivo study, statistical differences in tumor growth were evaluated using a multiple comparisons of means by Tukey contrast, analyzed with the R v.2.14.1 software, applying a linear mixed-effect model using the Ime4 package. *p* < 0.05 was taken as the level of significance.

## 3. Results

### 3.1. OncomiRs Downregulation Has a Strong Impact on Adenoviral Oncolysis 

Genome-wide miRNA profiling of PDAC samples identified the deregulation of several miRNAs [22]. We selected three of the most highly upregulated miRNAs, with well-studied OncomiR functions: miR-21, miR-93, and miR-222 [5,23,24] (Table 1). 

To analyze the influence of miRNAs on adenoviral cytotoxicity, we transduced PANC-1 and MIA PaCa-2 cells with three different lentiviral vectors delivering the Cas9 and the corresponding sgRNA and generated knockout (KO) pools of cells for the three miRNAs (miR-21 KO, miR-93 KO, and miR-222 KO). In parallel, PANC-1 and MIA PaCa-2 control cells were obtained by lentiviral transduction with a scrambled sgRNA for each miRNA (PANC-1 Ctrl, MIA PaCa-2 Ctrl). Following puromycin selection, the expression analysis of targeted miRNAs revealed residual or almost undetectable levels of miR-21, miR-93, or miR-222 in the two cell lines (Figure S1A). To have a null miRNA background we obtained single clones with an undetectable expression of miR-21 and miR-93 (Figure S1B). Genetic alterations in the miRNA regions were confirmed by DNA sequencing, detecting indel mutations in all the cellular models (Figure S1C). 

**Figure 1:**
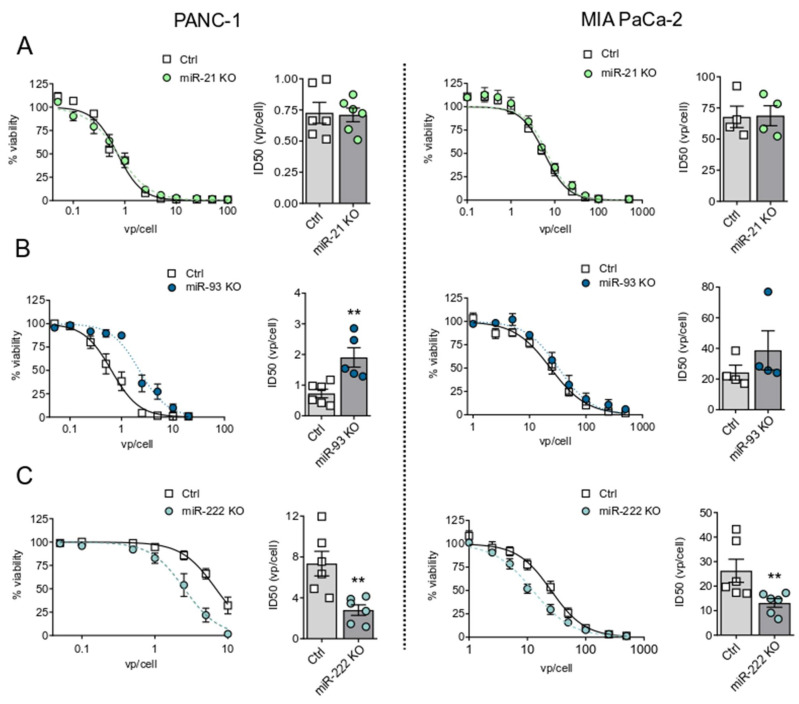
Adenoviral cytotoxicity in miR-21, miR-93, and miR-222 KO PANC-1 and MIA PaCa-2 cell lines. Control and miR-21 KO (**A**), miR-93 KO (**B**), and miR-222 KO (**C**) cells were infected with a dose range of Adwt. Cell viability was measured at 7 days post-infection (PI) by MTT assay, and infectious dose (ID50) values were determined. Data are shown as mean ± SEM for at least 3 independent biological replicates. Significance was assessed using a two-tailed Mann-Whitney test. ** *p* < 0.01.

To evaluate the effects of the miRNA downregulation on adenoviral activity, we infected the different cell lines with a battery of wild-type adenoviral doses and analyzed the cytotoxicity 7 days later. MIA PaCa-2 and PANC-1 miR-21 KO pools, expressing residual levels of miR-21, resulted in an adenoviral cytotoxic response similar to control cells. However, miR-21KO 1/2 cells, derived from individual clones, with absent miR-21 levels, displayed an extremely reduced adenoviral cytotoxicity, highlighting the sensitivity of the system to miR-21 content (Figure 1A, Figure S2). The evaluation of miR-93 influence in adenoviral response showed that miR-93 KO cells displayed extremely low sensitivity to adenoviral cytotoxicity, with an ID50 that doubled that of control cells (Figure 1B). Interestingly, inhibition of miR-222 expression increased the sensitivity to adenoviral cell death both in PANC-1 and MIA PaCa-2 cells, with 2.5-fold and 2-fold decreased ID50 values, respectively (Figure 1C). 

**Figure 2:**
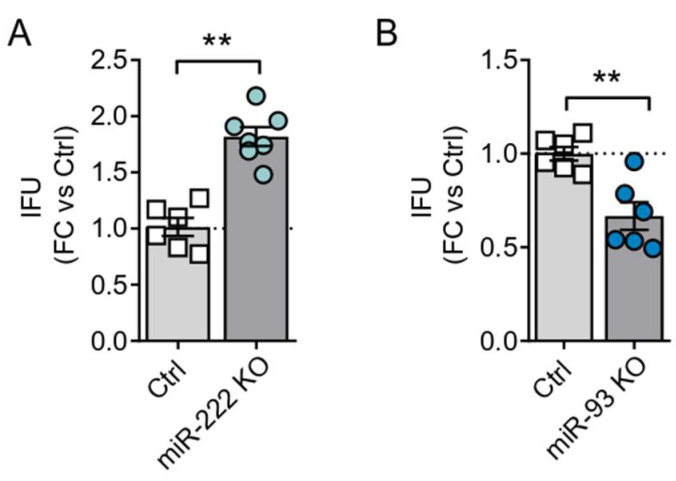
Viral particles release in miR-222 KO and miR-93 KO cells. PANC-1 Ctrl and KO for miR-222 (**A**) and miR-93 (**B**) were seeded in triplicate and infected at 1 IFU/cell with Adwt. At 72 h PI, supernatants were collected, and viral particles quantified by qPCR using specific primers for the adenoviral hexon gene. Data are shown as mean ± SEM for at least four independent biological replicates and represented as fold change with control cell lines. Significance was assessed using a two-tailed Mann-Whitney test. ** *p* < 0.01.

To further characterize the effects of miR-222 inhibition in adenoviral oncolysis, we assessed the production and consecutive release of viral particles. For this, PANC-1 Ctrl and miR-222 KO cells were infected with Adwt, and the viral yield was analyzed at 72 h post-infection. Quantification of viral genomes in the supernatant of infected cells revealed an increase in the adenoviral particles release by miR-222 KO cells, further supporting the idea that miR-222 inhibition facilitates adenoviral oncolysis (Figure 2A). In line with the cytotoxic experiments, miR-93 KO cells displayed reduced released of viral particles (Figure 2B).

MiR-222 is in a cluster region with miR-221, separated only by 727 bp; the two miRNAs share the same promoter and common transcriptional regulators, resulting in similar alteration profiles. To confirm whether the effects of increased sensitivity to adenoviral oncolysis observed in miR-222 KO cells were specific for miR-222, and independent of miR-221, we assessed miR-221 expression in miR-222 KO cells. We observed similar levels between control and KO cells, suggesting that the proviral effects were specific for miR-222, and confirming that our CRISPR KO only affected miR-222 expression (Figure S3A).

### 3.2. miR-222 Sponges Engineered in the Oncolytic Adenovirus AdNuPAR-E-miR222-S Reduce miR-222 Levels and Regulate Target Genes That Facilitate Viral Production

To evaluate the advantages of miR-222 inhibition on oncolytic potency in a more translational approach, we assessed the introduction of artificial miR-222 sponges in an oncolytic adenoviral backbone, the AdNuPARmE1A [11]. miRNA sponges are a recently developed technology consisting of constructs containing multiple artificially engineered miRNA binding sites. Following the introduction of these sequences inside the cell, the sponges recognize their miRNA target and, upon binding, saturate its ability to regulate natural mRNAs [14,15]. 

Since miR-222 and miR-221 share the same seed sequence, very likely the inhibition of miR-222 by a sponge strategy will also lead to downregulation of miR-221. Thus, we checked the impact of miR-221 inhibition on adenoviral activity. With this aim, PANC-1 miR-221 KO cells were generated (Figure S3B) and infected with a battery of Adwt doses. Cytotoxic analysis revealed similar dose-response curves between control and miR-221 KO-infected cells, indicating that miR-221 inhibition was not modifying adenoviral activity (Figure S3C).

**Figure 3:**
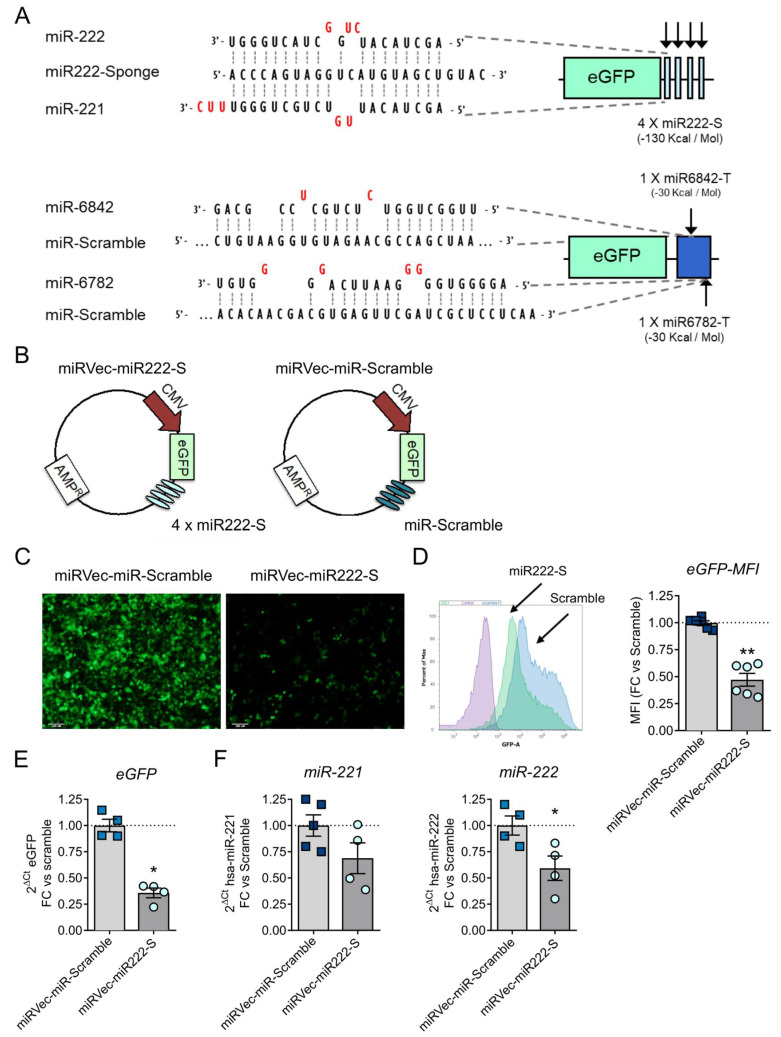
Generation and functional validation of miR-222 sponge in miRVec-eGFP-miR222-S and miRVec-eGFP-Scramble. (**A**). Scheme of the sponge design with a sequence alignment of miR-222, miR-221, and the sponge (upper figure). A representation of the Scramble sequence with predictive off-targets miRNAs is also provided (lower figure). (**B)**. Schematic representation of miRVec-eGFP-miR222-S and miRVec-eGFP-Scramble constructs. (**C**–**F**). Expression of eGFP from HEK-293T cells transfected with miRVec-eGFP-miR222-S or miRVec-eGFP-Scramble. (**C**). Representative images of eGFP fluorescence (scalebar 100 µm). (**D**). eGFP quantification by flow cytometry. The left panel shows a representative plot. The right panel shows the quantification of mean fluorescence intensity (MFI). (**E**). eGFP quantification by qRT-PCR. (**F**). Quantification of miR-221 and miR-222 content in HEK-293T cells transfected with miRVec-eGFP-miR222-S and miRVec-eGFP-Scramble. Values are represented as fold change compared to the Scramble construct. Data are shown as mean ± SEM for at least four independent biological replicates. Significance was assessed using a two-tailed Mann–Whitney test. * *p* < 0.05, ** *p* < 0.01.

For the sponge design, we chose 4 tandem repeats of miRNA binding sites, separated by 4 random nucleotides working as spacers [15,27,28]. Each repeat consisted of a sequence of 21 nucleotides, having a bulged region from nucleotides 9 to 12, flanked by two extremities of 9 and 8 nucleotides with perfect complementarity to miR-222 (miR222-S). A sponge control sequence was obtained as well, by scrambling miR222-S nucleotides (miR-Scramble). Both miR222-S and miR-Scramble were generated with the help of miRNAsong software (Figure 3A) [29]. To evaluate their activity, we cloned miR222-S and miR-Scramble in the 3’UTR of the eGFP gene, controlled by the CMV promoter inside a miRVec vector, generating the plasmids miRVec-miR222-S and miRVec-miR-Scramble (Figure 3B). HEK293T cells were transfected with each of the plasmids, and eGFP expression was monitored as a measure of sponge activity. Cells transfected with miRVec-miR-222-S revealed a significant reduction in eGFP expression, analyzed by flow cytometry and mRNA transcript quantification (Figure 3C–E), suggesting the binding of cellular miR-222 to the sponge. The sponge functionality was confirmed by the quantification of endogenous miR-222/miR-221 levels, observing a reduction of both miR-222 and miR-221 of 50% and 30%, respectively (Figure 3F).

**Figure 4:**
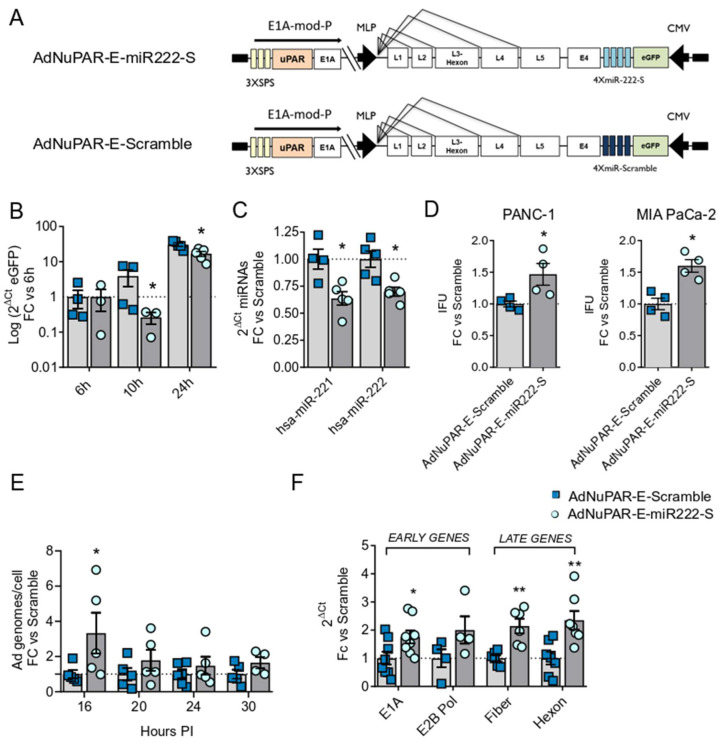
AdNuPAR-E-miR222-S exerts sponge effects over its target miRNAs and displays improved adenoviral fitness. (**A**) Schematic representation of the adenoviral backbones containing the sponge or scramble sequence. (**B**) qRT-PCR of eGFP at the indicated time-points from PANC-1 cells infected with 10 IFU of AdNuPAR-E-miR-222-S or AdNuPAR-E-Scramble. (**C**) q-RT-PCR of miR-221 and miR-222 content in PANC-1 cells at 24 h after infection PDAC cells. (**D**) PANC-1 or MIA PaCa-2 cells were infected with 1 IFU/cell or 5 IFU/cell of either virus, respectively. Seventy-two hours later supernatants were collected and the number of viral particles detected by qRT-PCR quantification of the adenoviral hexon gene. (**E**) Quantification of the intracellular viral genomes at the indicated times PI of PANC-1 cells infected with 2.5 IFU/cells of AdNuPAR-E-Scramble or AdNuPAR-E-miR222-S. Values are represented as adenoviral genomes/cells, with respect to viral genomes at 4h. (**F**) Quantification of E1A, E2B Pol, Fiber and Hexon mRNA levels at 30 h PI in PANC-1 cells infected with 10 IFU/cell of either virus. Data are shown as mean ± SEM for at least four independent biological replicates. Significance was assessed using a two-tailed Mann–Whitney test. * *p* < 0.05, ** *p* < 0.01.

Next, we generated the tumor-selective replicative adenoviruses AdNuPAR-E-miR222-S and the Ad-NuPAR-E-Scramble, by insertion of the CMV-eGFP-miR222-S or the scrambled sequence between the E4 and the ITR regions of the AdNuPARmE1A genome (Figure 4A). In this virus, tumor selectivity is provided by the combination of the uPAR promoter with the NOTCH-responsive elements regulating E1A [11]. To demonstrate the decoy effect of the oncolytic adenovirus-delivered sponge, PANC-1 cells were infected with 10 IFU of AdNuPAR-E-miR222-S or AdNuPAR-E-Scramble, and eGFP expression was analyzed at different time-points. AdNuPAR-E-miR222-S displayed reduced eGFP expression at 10h and 24h post-infection, in line with miR-222 sponge effect (Figure 4B). Furthermore, PANC-1 cells infected with AdNuPAR-E-miR222-S displayed significantly reduced miR-222 and miR-221 levels when compared to AdNuPAR-E-Scramble infected cells (Figure 4C).

We next evaluated whether inhibition of miR-222 by AdNuPAR-E-miR222-S was impacting viral production. For this, PANC-1 and MIA PaCa-2 cells were infected with AdNuPAR-E-miR222-S or AdNuPAR-E-Scramble, and the release of viral particles was analyzed three days later. Infection of cells with AdNuPAR-E-miR222-S led to a 1.5-fold higher virions release compared to AdNuPAR-E-Scramble (Figure 4D).

To assess whether the increase in viral particle release could be the consequence of enhanced viral genome replication, PANC-1 cells were infected with AdNuPAR-E-miR222-S or AdNuPAR-E-Scramble, and intracellular viral genomes were quantified at different hours of infection. At all the time-points analyzed, cells infected with AdNuPAR-E-miR222-S showed a higher number of intracellular viral genomes when compared to cells infected with the Scramble virus. The effects were highly evident at 16 hours after infection, reaching statistical significance (Figure 4E). These results suggested that miR-222 inhibition could promote the viral lytic cycle by means of facilitating viral DNA replication. To further characterize the effects of miR-222 inhibition on adenoviral biology, we monitored the expression of early (E1A and E2B Pol) and late (fiber and hexon) transcripts in PANC-1 infected cultures (Figure 4F). Increased mRNA levels were detected following AdNuPAR-E-miR222-S infection, indicating that miR-222 could regulate pathways affecting the transcription of viral genes.

**Figure 5:**
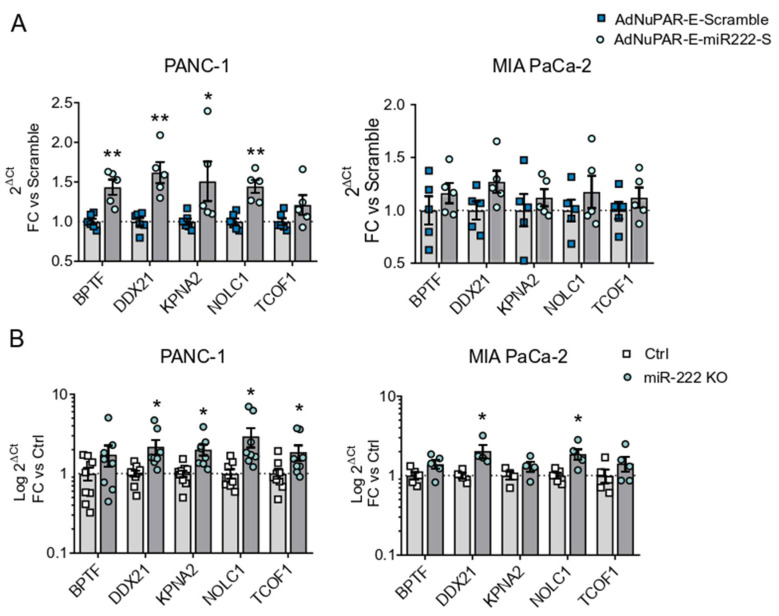
Effects of miR-222 depletion on the expression of selected miR-222 target genes. (**A**) qRT-PCR expression analysis of bioinformatically identified miR-222 target genes in PANC-1 or MIA PaCa-2 cells 24 h PI with 10 IFU/cell of AdNuPAR-E-Scramble or AdNuPAR-E-miR222-S. (**B**) qRT-PCR expression analysis of bioinformatically identified miR-222 target genes in Ctrl and CRISPR miR-222 PANC-1 KO and MIA PaCa-2 KO cells. Data are shown as mean ± SEM for at least five independent biological replicates. Significance was assessed using a two-tailed Mann-Whitney test. * *p* < 0.05, ** *p* < 0.01.

To provide some insight into the mechanisms boosting adenoviral fitness promoted by miR-222 inhibition, we performed a bioinformatic study to identify putative miR-222 target genes. We hypothesized that in PDAC cells the upregulated miR-222 will maintain low levels of genes that are relevant to the complete adenoviral life cycle. Genes predicted to be regulated by miR-222, from the list of upregulated genes after an adenoviral infection described by Reyes and collaborators [21] were identified. From this list, five genes were selected: Bromodomain PHD Finger Transcription Factor (BPTF), Nuclear RNA Helicase 2 (DDX21), Karyopherin Subunit Alpha 2 (KPNA2), Nucleolar and coiled-body phosphoprotein 1 (NOLC1), and Tetracle Ribosome biogenesis factor 1 (TCOF1) [30,31,32]. We analyzed the expression of the 5 genes in PANC-1 and MIA PaCa-2 cells infected with AdNuPAR-E-miR222-S or Ad-NuPAR-E-Scramble. Interestingly, we observed significantly increased levels in 4 out of the 5 genes in the PANC-1 cultures infected with the miR-222 sponge-adenovirus. MIA PaCa-2 cells showed a similar tendency, although this trend did not reach statistical significance (Figure 5A). To gather further evidence of the relationship between the expression of the indicated genes and miR-222 inhibition, we analyzed the mRNA levels of the target genes in PANC-1 and MIA PaCa-2 miR-222 KO cells. Again, higher amounts of transcripts were detected for the 5 genes in the miR-222 KO cells (Figure 5B). In line with the molecular functions of the selected genes modulating transcription (BPTF, DDX21), protein translation (NOLC1, TCOF1), or nuclear import (KPNA2) it is feasible to speculate that restoring their cellular content in cancer cells could facilitate viral production.

### 3.3. Inhibition of miR-222 by miR-222 Sponges Displayed Enhanced In Vitro Cytotoxicity and Improved Antitumoral Activity

**Figure 6:**
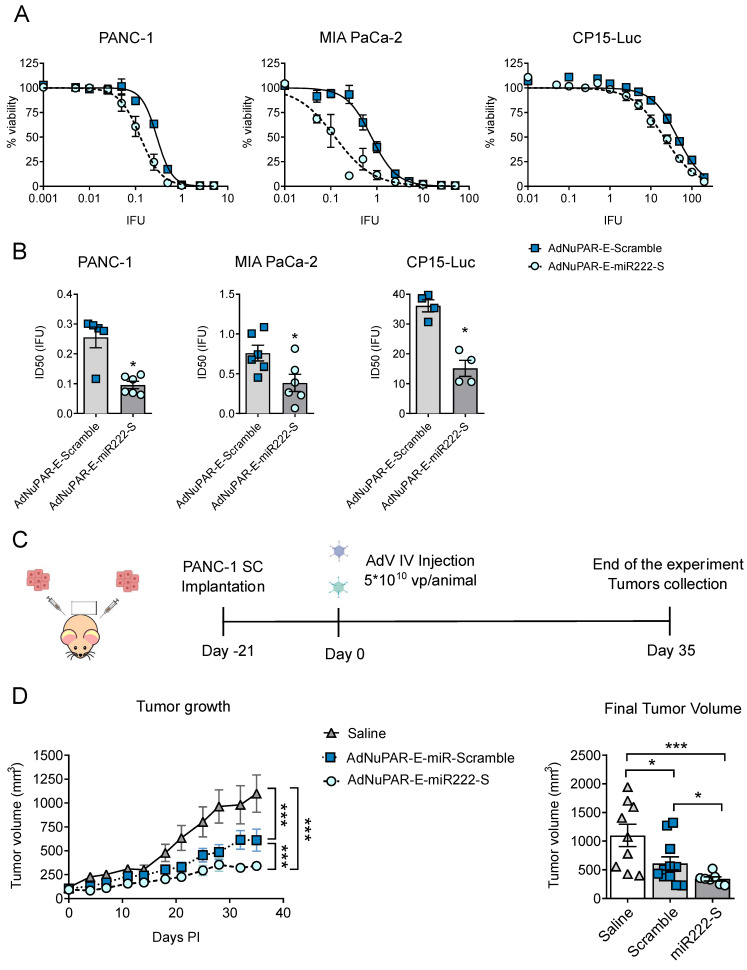
AdNuPAR-E-miR222-S oncolytic potency in vitro and antitumor activity in vivo. (**A**) Viability Curves in PANC-1, MIA PaCa-2, and CP15-Luc cell lines. Cells were infected with AdNuPAR-E-Scramble or AdNuPAR-E-miR222-S at the indicated viral doses and cell viability was measured 7 days later by an MTT assay. (**B**) ID50 values for the three PDAC cell lines. Values are expressed as mean ± SEM of at least four independent biological replicates. (**C**) Schematic representation of the in vivo experiment. (**D**) Tumor growth follow-up. Left panel shows growth curves for the different treatments. Right panel shows mean tumor volumes on day 35 for each group. Data are shown as mean ± SEM. (*n* ≥ 8 tumors/treatment group). Statistical differences in tumor growth (**D**) were evaluated using a multiple comparison of means by Tukey contrast, analyzed with the R v.2.14.1 software, applying a linear mixed-effect model using the Ime4 package. *p* < 0.05 was taken as the level of significance. For the remaining graphs significance was assessed using a two-tailed Mann-Whitney test. * *p* < 0.05, *** *p* < 0.001.

To further exploit the therapeutic potential of AdNuPAR-E-miR222-S we studied the cytotoxicity of the new virus and compared it to the scramble control. Three different pancreatic cancer cell lines PANC-1, MIA PaCa-2, and CP15-Luc were infected with a battery of viral doses. AdNuPAR-E-miR222-S demonstrated to be more potent than the control virus in all the cell lines tested with 2,6-, 2- and 2,4-fold lower IC50 values respectively (Figure 6A,B Table 2). 

Next, we assessed the antitumor efficacy of AdNuPAR-E-miR222-S in a xenograft model of PDAC. Mice bearing PANC-1 tumors were intravenously injected with a single dose of saline, AdNuPAR-E-Scramble or AdNuPAR-E-miR222-S (Figure 6C). The two oncolytic adenoviruses controlled tumor progression, but, notably, inhibition of tumor growth promoted by AdNuPAR-E-miR222-S administration was consistently higher than that of the scrambled virus (Figure 6D, left panel). On day 35 after treatment, tumors were significantly smaller in the AdNuPAR-E-miR222-S group (Figure 6D, right panel).

Interestingly, systemic injection of AdNuPAR-E-miR222-S was safe, with no signs of major toxicity. Immunocompetent mice receiving i.v. AdNuPAR-E-miR222-S at 5 × 10^10^ vp/mice experienced weight loss, with a maximum at day 4 from which all the animals recovered by day 12 (Figure S4).

These data strongly suggest that miR-222 inhibition can control tumor progression in preclinical models of PDAC. With this attractive sponge approach, we propose a new technology, easy to handle, for improving adenoviral oncolysis and increasing the anticancer efficacy of viral therapies. 

## 4. Discussion

Therapies applying oncolytic adenoviruses as single agents against tumors have shown a limited response in clinic. Different studies have highlighted the role of miRNAs in the replication and propagation of viruses, together with the impact of perturbed miRNome in cancer cells, envisioning miRNAs as susceptible molecules to facilitate oncolysis [10,33]. We show that interfering with the expression of selected upregulated OncomiRs in pancreatic cancer cells impacts adenoviral oncolysis. We observed that hindrance of miR-21 and miR-93 expression hampered virus replication, whereas inhibition of miR-222 facilitated adenoviral oncolysis. The impaired adenoviral activity resulting from miR-93 and miR-21 blockade could be partially explained by their repressing activity on the interferon response. Under normal conditions, adenoviral cell entry stimulates a strong immune response converging on the activation of interferon-stimulated genes (ISGs), that try to counteract the viral propagation [34]. The absence of interferon signaling permits the activation of the E1A adenoviral gene transcription, which in turn triggers the expression of the remaining viral genes, promoting the adenoviral replication cycle [35]. c-GAS is a cytosolic protein acting as a first-line sensor for viral and microbial infections, which upon detection of exogenous dsDNA activates a signaling cascade that leads to the activation of ISGs, to restrain host invasion. miR-93 negatively regulates c-GAS, repressing the production of interferon and thus creating the conditions to facilitate viral propagation [36]. Accordingly, we speculate that in the context of miR-93 loss an upregulation of c-GAS signaling could take place, interfering with adenoviral replication. Moreover, a well-detailed study documented the upregulation of miR-93 at very early and late time-points of adenoviral infections, suggesting the need for miR-93 to successfully complete the adenoviral cycle [5]. Regarding miR-21 effects on viral infections, evidence showed that this miRNA has a pro-viral activity during Hepatitis C virus (HCV) infections. Upon cell entry, HCV responds to type I interferon inhibition through the upregulation of miR-21, which is responsible for the repression of MyD88 and IRAK1, adaptors proteins required for a successful IFN-1 signaling activation [37]. Thus, in the absence of miR-21, very likely the interferon pathway might hamper viral replication. This is in accordance with our results, where the complete lack of miR-21 will be detrimental, but small levels of miR-21 expression could be sufficient for the virus to propagate. 

Interestingly, we observed that miR-222 blockade boosted adenoviral activity. Of note, Ad5 infection causes a major reduction in miR-222, suggesting that adenovirus requires reduced levels of miR-222 to propagate [7]. Similarly, infections with the DNA viruses Epstein Barr Virus (EBV) and the Hepatitis B virus (HBV) also lead to miR-222 downregulation, suggesting that this miRNA may participate in common mechanisms exploited by DNA viruses [38,39]. In line with this, we observed that introduction of miRNA sponges in the oncolytic adenovirus AdNuPAR-E-miR222-S downregulated miR-222, increased viral yield, and improved adenoviral oncolysis. All properties exhibited by miR-222 made its inhibition a valid strategy to sensitize pancreatic cancer cells to adenoviral oncolysis. Exploring the mechanisms involved in the oncolysis potentiation induced by miR-222 inhibition, our study identified BPTF, DDX21, KPNA2, NOLC1, and TCOF1 as miR-222 target genes, carrying miR-222 binding sites in their 3'UTRs. The expression of the five genes was restored in cells infected with AdNuPAR-E-miR222-S, being the rescue of DDX21 and NOLC1 statistically significant in the two cell lines studied, thus suggesting an important contribution to viral activity. The DEAD-box RNA helicase (DDX21) is a multitasking enzyme with functions in RNA metabolism and coordination of transcriptional programs, regulating both steps in ribosome biogenesis and cooperative functions to resolve genomic R-loops [32,40]. Recently, the DDX21 function has been explored in the context of DNA virus replication, finding out that DDX21 was essential for human cytomegalovirus (HCMV) replication. The absence of DDX21 led to the accumulation of R-loops, which prevented viral late gene transcription and subsequently inhibited HCMV replication [41]. As a DNA virus, adenovirus can form R-loops following transcription of early and late adenoviral genes regulating gene expression [42]. In this context, we can hypothesize that our findings will be supporting a potential effect of DDX21 in adenovirus replication since the increased viral activity was associated with the restoration of DDX21 expression in AdNuPAR-E-miR222-S infected cells.

An additional contribution to increased adenoviral replication in cancer cells treated with the miR-222 sponge-virus can be derived from the NOLC1-TCOF1 complex-mediated effects. The treacle protein (TCOF1) is a regulator of RNA polymerase I that recruits the nucleolar and coiled-body phosphoprotein 1 (NOLC1) to sites of adenovirus replication in the nucleus of infected cells. The depletion of TCOF1 alters NOLC1 localization and in TCOF1 knockdown cells, there is a reduction of adenovirus late proteins, reduced viral genome accumulation, and diminished viral yield [21]. Our results evidenced that in the conditions of NOLC1-TCOF1 rescue after miR-222 adenoviral sponges there was increased genome accumulation, enhanced viral gene expression, and improved viral yields.

Collectively, miR-222 inhibition in AdNuPAR-E-miR222-S infected cells displays a plethora of cellular changes involving different mechanisms facilitating adenoviral replication, that provide the new oncolytic adenovirus with good antitumor properties. Of notice, the significant antitumor effects observed by AdNuPAR-E-miR222-S could also be explained by the mitigation of miR-222 pro-tumorigenic functions. In fact, we observed a marked rescue of the tumor suppressor genes, p57, and the phosphatase and tensin homolog PTEN, widely described as miR-222 targets, (Figure S5). 

## 5. Conclusions

Our study shows that miR-222 inhibition by the introduction of a miRNA sponge in adenovirus improves oncolysis by enhancing adenoviral fitness, and blocks OncomiR pathways resulting in improved antitumor efficacy in PDAC preclinical models. These studies support the rationale of AdNuPAR-E-miR222-S being an attractive combined strategy that incorporates the benefits of miR-222 inhibition and virotherapy in a single therapeutic product.

## Figures and Tables

**Table 1:** miRNAs expression represented as Fold Change (FC) expression in PDAC tumors compared to a healthy pancreas.

miRNA	FC vs. Healthy	Ref
hsa-miR-21	11.18	[23]
hsa-miR-93	2.75	[23]
hsa-miR-222	2.70/32	[25,26]

**Table 2:** ID50 values of AdNuPAR-E-222-S and AdNuPAR-E-Scramble in different PDAC cell lines.

Cell Line	AdNuPAR-E-Scramble (IFU ± SEM)	AdNuPAR-E-miR222-S (IFU ± SEM)
PANC-1	0.255 ± 0.035	0.096 ± 0.0124
MIA PaCa-2	0.758 ± 0.099	0.385 ± 0.108
CP-15 Luc	36.110 ± 2.039	15.132 ± 2.674

## Data Availability

The data presented in this study are available on request from the corresponding author.

## Supplementary Materials

The following are available online at https://www.mdpi.com/article/10.3390/cancers13133233/s1, Figure S1: Characterization of miRNA CRISPR KO cell lines, Figure S2: Sensitivity of PANC-1 miR21KO clones to adenoviral infections, Figure S3: miR-221 response to adenoviral oncolysis, Figure S4: Follow-up of body weight, Figure S5: p57 and PTEN gene expression in PANC-1 xenografts. Table S1: List of sgRNAs used in the study, Table S2: List of primers used for sequencing and cloning processes, Table S3: List of ssDNA used for the construction of miRNA sponges, Table S4: List of primers used for gene expression analysis via qRT-PCR.
