# Supplementary material for: Inhibition of miR-222 by Oncolytic Adenovirus-Encoded miRNA Sponges Promotes Viral Oncolysis and Elicits Antitumor Effects in Pancreatic Cancer Models
Source: Cancers (Basel). 2021 Jun 28;13(13):3233. doi: 10.3390/cancers13133233 (PMC8267801; doi:10.3390/cancers13133233)
Supplement: Supplementary file 1 [file cancers-13-03233-s001.zip › cancers-1256222-supplementary.pdf]

Inhibition of miR-222 by Oncolytic Adenovirus-Encoded miRNA Sponges Promotes Viral Oncolysis and Elicits Antitumor Effects in Pancreatic Cancer Models

Giulia Raimondi, Sabrina Gea-Sorlí, Marc Otero-Mateo, Cristina Fillat

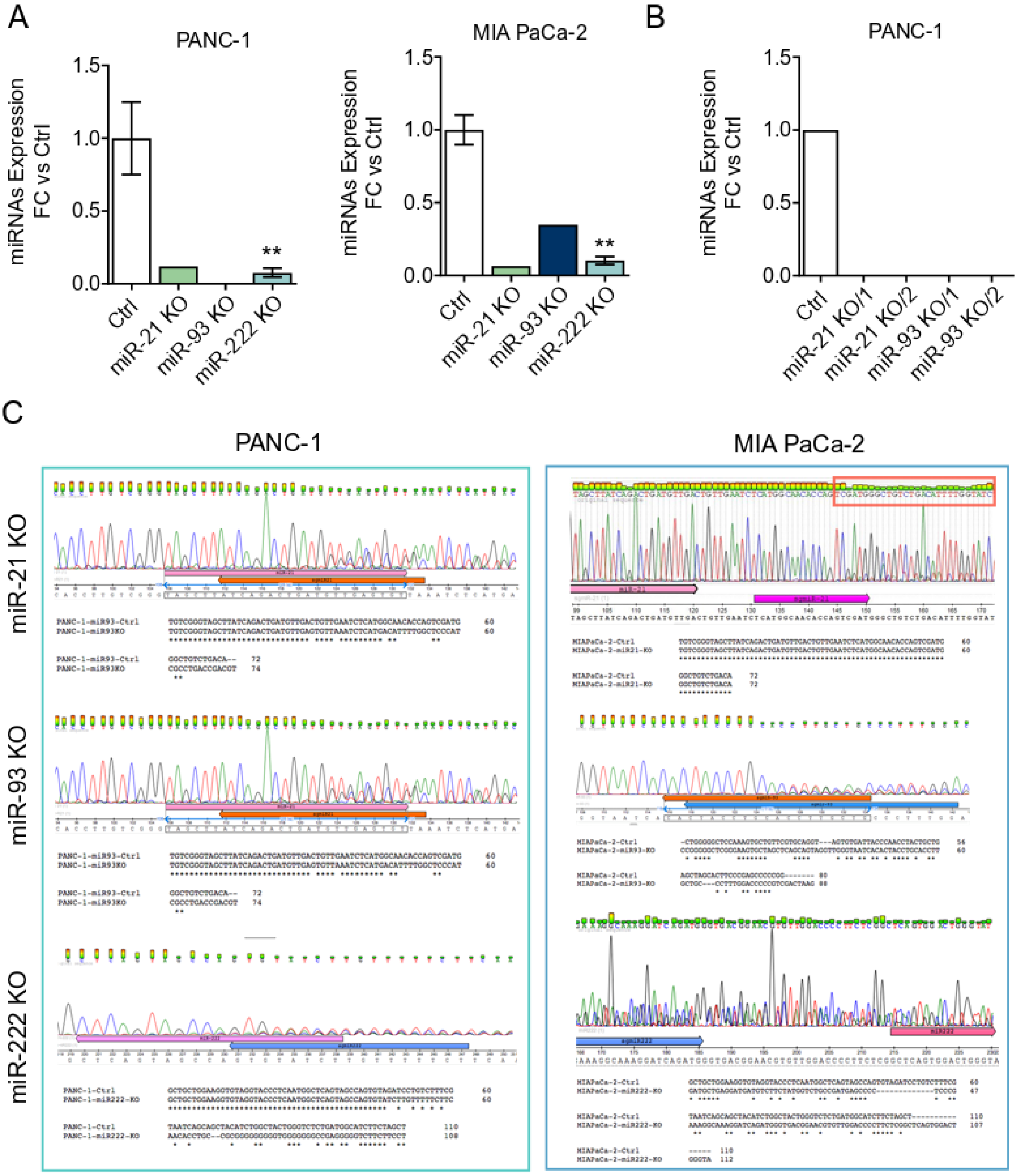

Figure S1. Characterization of miRNA CRISPR KO cell lines. **A-B** miR-21, miR-93 and miR-222 expression in PANC-1 and MIA PaCa-2 KO cell lines pools (**A**) or isolated clones (**B**). Values are represented as Fold Change (FC) respect to Ctrl cells. **C**. DNA sequences of miR-21, miR-93, miR-222 genomic regions in KO cells annealed with WT sequence. Sequence mismatches were detected in the miRNA sequences, for all KO cells except for MIAPaCa-2 miR-21 KO in which the mismatches were shown in sequences flanking the miRNA.

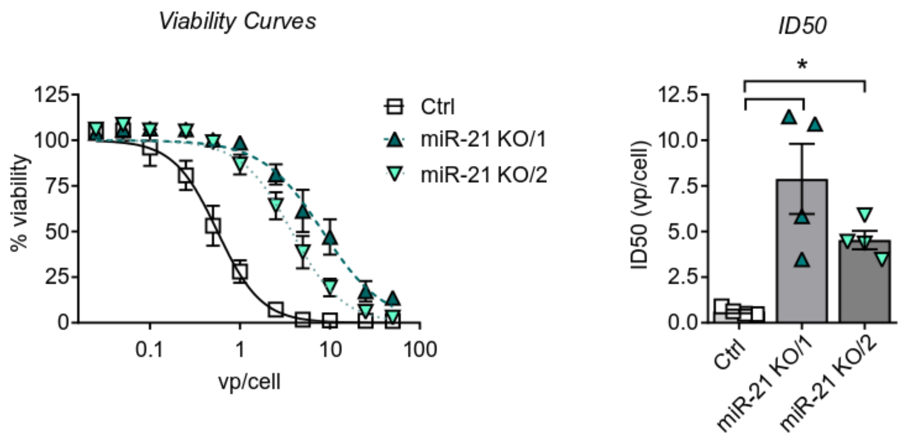

Figure S2. Sensitivity of PANC-1 miR-21KO clones to adenoviral infections. PANC-1 Ctrl cells and miR-21 clones KO/1 and KO/2 were infected with a battery of Adwt doses. Cell viability was measured 7 days post infection (PI) by MTT assay, and infectious dose (ID50) values determined. Data are shown as mean ± SEM for at least 3 independent biological replicates. Significance was assessed using a two-tailed Mann-Whitney test. *$p < 0.05$.

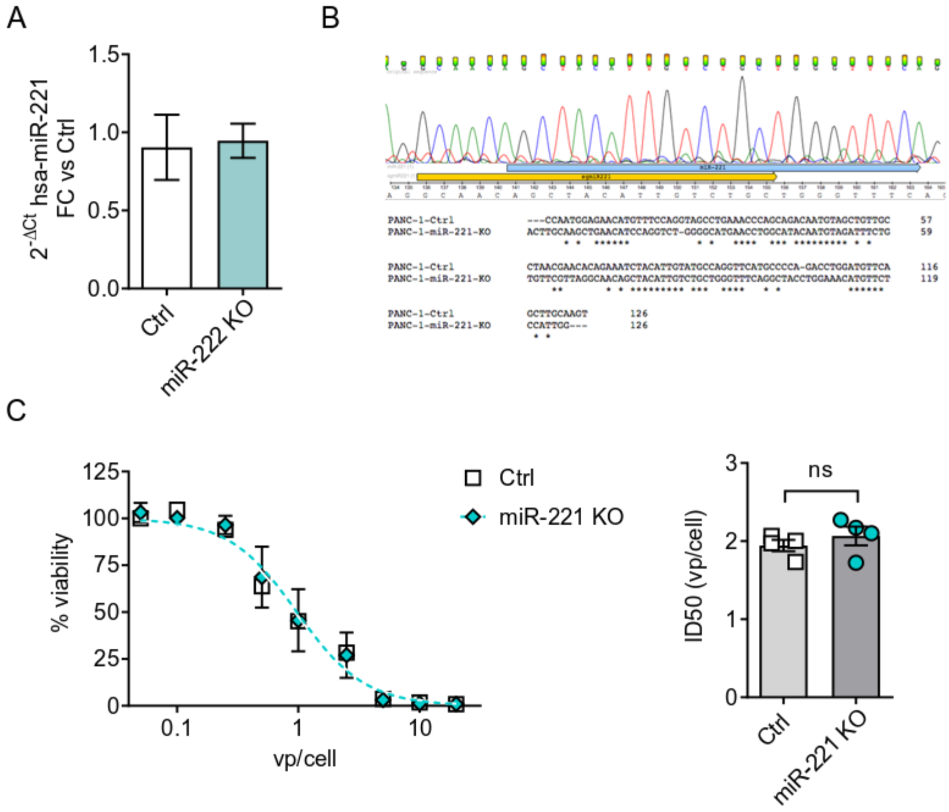

Figure S3. miR-221 response to adenoviral oncolysis. A. miR-221 expression in Ctrl and miR-222 KO cells, results are represented as Fold Change with Ctrl cells. **B**. DNA sequence alignment of miR-221 genomic region in miR-221 KO cells compared with control cells. **C**. PANC-1 Ctrl and miR221 KO cells infected with a battery of Adwt doses. Cell viability was measured 7 days PI by MTT assay, and ID_{50} values were determined. Data are shown as mean ± SEM for at least 3 independent biological replicates. Significance was assessed using a two-tailed Mann-Whitney test.

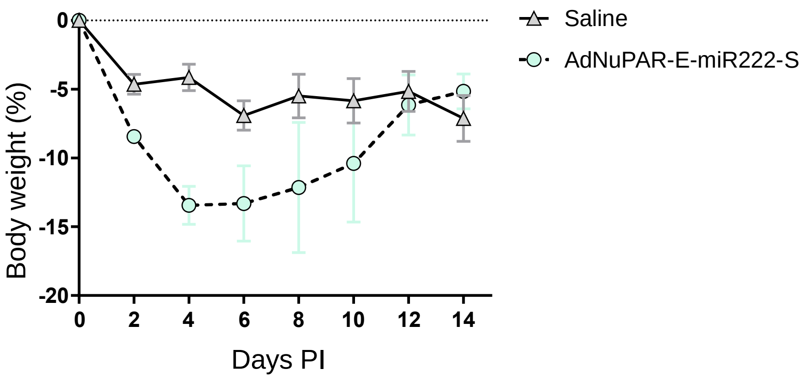

Figure S4. Follow-up of body weight. Percentage of body weight variation in immunocompetent C57Bl/6 mice after intravenous administration of saline solution or AdNuPAR-E-miR222-S at 5 10^{10}vp/animal.

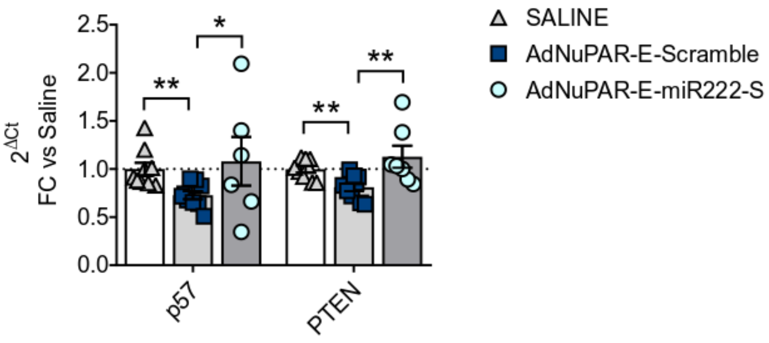

Figure S5. p57 and PTEN gene expression in PANC-1 xenografts. qPCR analysis of p57and PTEN mRNA in PANC-1 tumors treated with saline, AdNuPAR-E-Scramble or AdNuPAR-E-miR222-S at 5 10^{10}vp/animal ($n \geq 6$ tumors/group). mRNA values were relativized to GAPDH expression in each replicate and represented as FC to saline treated. Data are shown as mean ± SEM for at least 6 values. Significance was assessed using a two-tailed Mann-Whitney test. *$p < 0.05$, **$p < 0.01$.

Table S1. List of sgRNAs used in the study.

Targeted miRNA	sgRNA Fw (5′ → 3′)	sgRNA Rv (5′ → 3′)
hsa-miR-21	caccgTCATGGCAACACCAGTCGAT	aaacATCGACTGGTGTTGCCATGAc
hsa-miR-93	caccgCTCCAAAGTGCTGTTCGTGC	aaacGCACGAACAGCACTTTGGAGc
hsa-miR-221	caccGCAACAGCTACATTGTCTGC	aaacGCAGACAATGTAGCTGTTGC
hsa-miR-222	caccgACGAAAGACAGGATCTACAC	aaacGTGTAGATCCTGTCTTTCGTc

Table S2. List of primers used for sequencing and cloning processes. For the primers set miRNA_Tails, small letters represent homology with adenoviral E4 regions and capital letters represent CMV promoter recognizing sequences.

Primer set	Primer Fw (5′ → 3′)	Primer Rv (5′ → 3′)	Application
miR21KO_Val	CCACACTCTGTCGTATCTGTG	AAGTGCCACCAGACAGAAGG	Sequencing
miR93KO_Val	TGAGGGAGACCAGACCCTTT	TTCTGCTTCCCCATGAACCT	Sequencing
miR-221KO_Val	GTCCCAGCATTTCTGACTGTTGG	GGCTTGTGGGTGCTATGCCTTC	Sequencing
miR-222KO_Val	GCTGGATCTCCAGCACCTAAG	CCCAAGCCCCAGCTGATAATG	Sequencing
LCRISPR_Val	GAGGGCCTATTTCCCATGATT	GGAGTGGGAATTGGCTCCGGTGCCC	Sequencing
miRVec_Val	CATGGTCCTGCTGGAGTTCG	CTTGCCAAACCTACAGGTGG	Sequencing
AdV_Val	CCAGAAACGAAAGCCAAAAA	TAATGAGGGGGTGGAGTTTG	Sequencing
miRNA_Tails	gaaaactacaattcccaacacatacaagttactccgccctaa<br>CTTTTATTTTATCGAATCTGC	cgtggcgcggggcgtgggaacggggcgggtgacgtaggtt<br>ACATTGATTATTGACTAG	Cloning

Table S3. List of ssDNA used for the construction of miRNA sponges. Bold sequences represent EcoRI restriction sites, red sequences represent extra stop codons inserted.

ssDNA for sponges	Sponge sequence	Final Primer sequence
Primer Scramble Fw	GTTCTTGCTGCACACGTAGCTGTAAGGT GTAGAACGCCAGCTAACGTAGTGGATC TCATGTACACAACGACGTGAGTTCGATC GCTCCTCAA	**AATTC**TAATGAGTTCTTGCTGCACACGT AGCTGTAAGGTGTAGAACGCCAGCTAA CGTAGTGGATCTCATGTACACAACGAC GTGAGTTCGATCGCTCCTCAA**G**
Primer Scramble Rv	TTGAGGAGCGATCGAACTCACGTCGTT GTGTACATGAGATCCACTACGTTAGCTG GCGTTCTACACCTTACAGCTACGTGTGC AGCAAGAAC	**AATTC**TTGAGGAGCGATCGAACTCACG TCGTTGTGTACATGAGATCCACTACGTT AGCTGGCGTTCTACACCTTACAGCTACG TGTGCAGCAAGAAC**TCATTAG**
Primer miR222-S Fw	ACCCAGTAGGTCATGTAGCTGTACACC CAGTAGGTCATGTAGCTGCATACCCAGT AGGTCATGTAGCTCGTAACCCAGTAGG TCATGTAGCT	**AATTC**TAATGAACCCAGTAGGTCATGT AGCTGTACACCCAGTAGGTCATGTAGCT GCATACCCAGTAGGTCATGTAGCTCGTA ACCCAGTAGGTCATGTAGCT**G**
Primer miR222-S Rv	AGCTACATGACCTACTGGGTTACGAGCT ACATGACCTACTGGGTATGCAGCTACAT GACCTACTGGGTGTACAGCTACATGAC CTACTGGGT	**AATTC**AGCTACATGACCTACTGGGTTAC GAGCTACATGACCTACTGGGTATGCAG CTACATGACCTACTGGGTGTACAGCTAC ATGACCTACTGGGT**TCATTAG**

Table S4. List of primers used for gene expression analysis via qRT-PCR.

Target Gene	Primer Fw (5′ → 3′)	Primer Rv (5′ → 3′)
Ad Hexo 1/2	GCCGCAGTGGTCTTACATGCACATC	CAGCACGCCGCGGATGTCAAAG
ALB	GCTGTCATCTCTTGTGGGCTGT	GGCTATCCAAACTCATGGGAG
BPTF	CGAAAAGGAGGAATCCGAGAGG	CGTAACATCAGGCTCACTCCAGC
DDX21	TCATCAAGGACGCACTATCATCT	CCTTTCAGGGTGATTTCCCTTT
EGFP	AAGATCCGCCACAACATCGA	AACTCCAGCAGGACCATGTG
HPRT	GATATAAGCCAGACTTTGTTGGATTTG	CTTGAACTCTCATCTTAGGCTTTG
KPNA-2	CGTCTTCACAGATTCAAGAACAAGG	CCTCTTCAGCATCTGGTCATCC
NOLC-1	TTCCTGCGCGATAACCAACTC	CCTGTAACTTTCGCTCTGGGA
P57	CAAGAGGCTGCGGTGAGC	GGACCGTTCATGTAGCAGCAACC
PTEN	CATACCAGGACCAGAGGAAACC	CGTCAAATCCAGAGGCTAGCAG
TCOF-1	CGATAACCAACTCTCAGAGGTGG	CTAAGAGGGAAGAGGCATTGG
